# Synthesis of 6,13-difluoropentacene

**DOI:** 10.3762/bjoc.16.181

**Published:** 2020-09-02

**Authors:** Matthias W Tripp, Ulrich Koert

**Affiliations:** 1 Department of Chemistry, Philipps-Universität Marburg, Hans-Meerwein-Straße 4, 35034 Marburg, Germany

**Keywords:** fluorinated acenes, Friedel–Crafts reaction, *ortho*-lithiation, synthesis

## Abstract

6,13-Difluoropentacene was synthesized from 1,4-difluorobenzene. Friedel–Crafts annulation of the latter with phthalic anhydride and subsequent reduction of the anthraquinone gave 1,4-difluoroanthracene. After *ortho*-lithiation and reaction with phthalic anhydride a carboxylic acid was obtained whose Friedel–Crafts acylation and subsequent reductive removal of the oxygen-functionalities resulted in the formation of the target compound. The HOMO–LUMO gap of 6,13-difluoropentacene was determined via UV–vis spectroscopy and compared to other fluorinated pentacenes.

## Introduction

**Figure 1:**
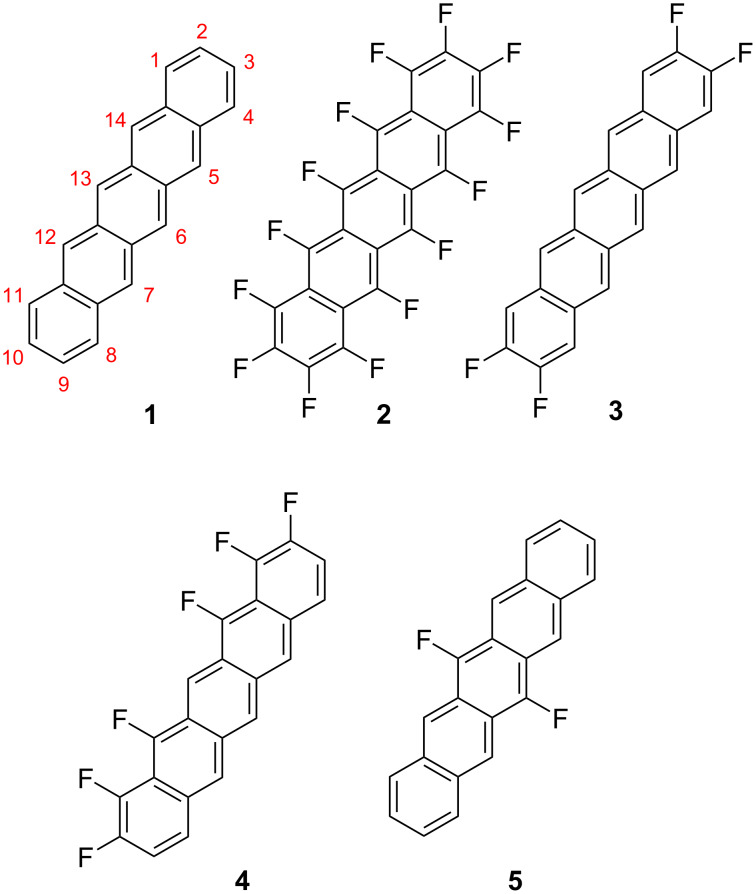
Structures of pentacene and fluorinated pentacenes.

Pentacenes are a prototype in the field of organic semiconductors due to their expanded π-systems and low HOMO–LUMO gaps with applications in different molecular-based organic electronics like OFETs, OLEDs or organic photovoltaics [1–3]. An advantage of organic molecular electronics over inorganic alternatives is the versatility in design of new molecular materials by derivatization of known compounds [4]. Since the performance of these devices is strongly dependent on the optoelectronic solid-state properties of the pentacenes, predictable control of the electronic structure is desirable. A common strategy to influence the electronic properties of pentacenes, without significant effect on the molecular shape, is fluorination [5]. Suzuki et al*.* showed in 2004 the strong effect of perfluorination on the electronic properties [6]. While unsubstituted pentacene (**1**, PEN, Figure 1) is known to be a p-type semiconductor, the perfluorinated counterpart perfluoropentacene (**2**, PFP) showed an n-type behavior. In their synthesis and structural as well as electronic characterization of F4PEN **3**, Bettinger et al. showed that the electronic properties of pentacenes are not a simple linear function of the degree of fluorination [7–8]. They reported that the fluorination in the 2,3,9,10-positions resulted in a larger HOMO–LUMO gap compared to PEN, while PFP showed a smaller gap than PEN. Another example for a partially fluorinated pentacene is the F6PEN **4** [9]. Its unilateral substitution pattern leads to a disctinct electronic structure and a criss-cross packing motif, unlike the herringbone structure of **1**–**3**.

Here, the synthesis and characterization of 6,13-difluoropentacene (**5**, F2PEN) is reported. Since the central ring of pentacenes is known to be a weak spot (because of light-induced dimerization or oxidation) [2] the effects of fluorination in these positions are of interest.

## Results and Discussion

**Scheme 1:**
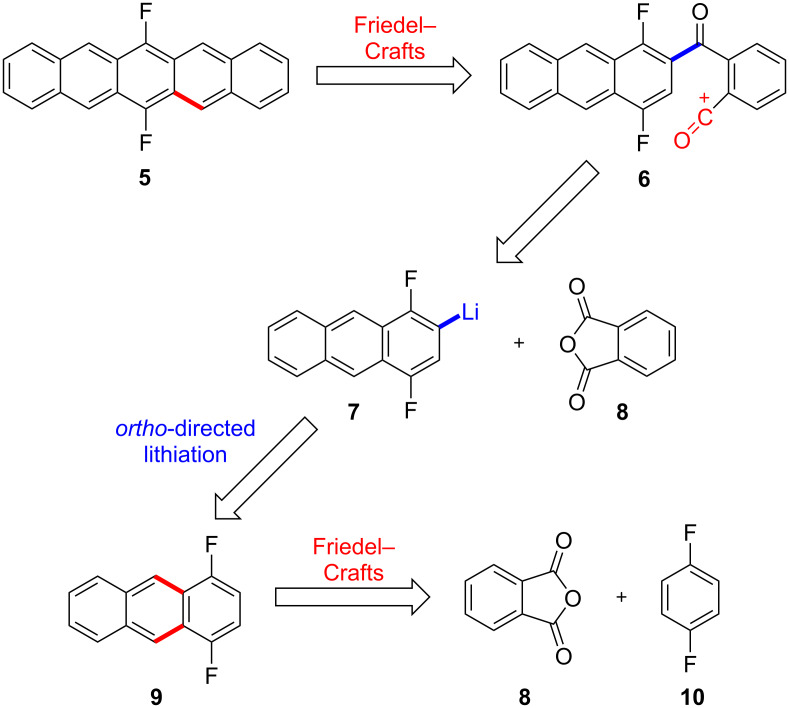
Retrosynthetic analysis of F2PEN **5**.

6,13-Disubstituted pentacenes are often synthesized starting from 6,13-pentacenedione [2–3,10–12]. Initial attempts to prepare 6,13-difluoropentacene (**5**) along this route failed [13]. Therefore, an alternative route that commences with the central ring already carrying the two fluorine substituents was investigated. The retrosynthetic analysis for this strategy is shown in Scheme 1. The formation of the C5,5a-bond colored in red could be accomplished by an intramolecular Friedel–Crafts type acylation with the acylium-cation intermediate **6**. The corresponding carboxylic acid precursor could be prepared by reaction of the anthracenyllithium **7** with phthalic anhydride (**8**)**.** Intermediate **7** could be accessed by *ortho*-lithiation of anthracene **9**. The synthesis of **9** by two consecutive Friedel–Crafts acylation reactions and reduction of the resulting anthraquinone could start from **8** and 1,4-difluorobenzene (**10**).

**Scheme 2:**
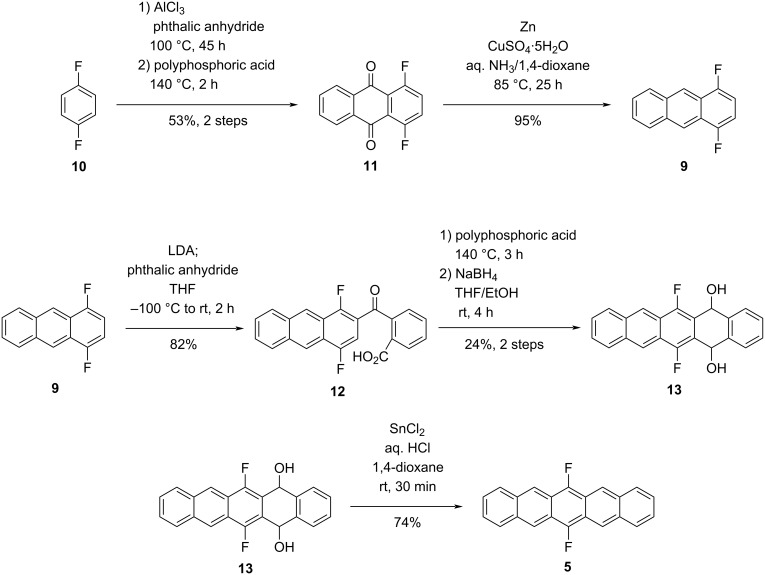
Synthesis of F2PEN **5**.

Starting point of the synthesis was the conversion of 1,4-difluorobenzene (**10**) to 1,4-difluoroanthraquinone (**11**, Scheme 2) [14]. The first Friedel-Crafts acylation of phthalic anhydride was achieved using AlCl_3_ in boiling 1,4-difluorobenzene (**10**). The second Friedel–Crafts acylation is then performed in polyphosphoric acid at 140 °C, giving anthraquinone **11** in 53% yield over two steps. Reduction of **11** to the anthracene **9** proceeded smoothly using zinc powder in 1,4-dioxane and aqueous ammonia under copper catalysis [15]. To achieve good yields, it was crucial to perform the reaction in a sealed pressure tube to prevent ammonia from degassing.

Different bases, temperatures and reaction times were screened to optimize the *ortho*-lithiation of **9**. The best results were achieved using LDA as a base at –78 °C for 15 min and then rapidly adding the lithiated species **7** to phthalic anhydride in THF precooled to –100 °C [16]. Using these conditions, the carboxylic acid **12** was isolated in 82% yield.

The subsequent Friedel–Crafts acylation proofed to be challenging. Using reagents like PCl_5_ resulted in a quantitative substitution of the fluorine substituents in the corresponding pentacenequinone by chloride. This side reaction could be avoided using polyphosphoric acid as Friedel–Crafts reagent. The pentacenequinone showed very low solubility, which complicated a chromatographic isolation. However, the crude ^1^H NMR showed sufficiently clean conversion, hence the subsequent reduction step using NaBH_4_ to diol **13** was performed without further purification of the quinone.

**Scheme 3:**
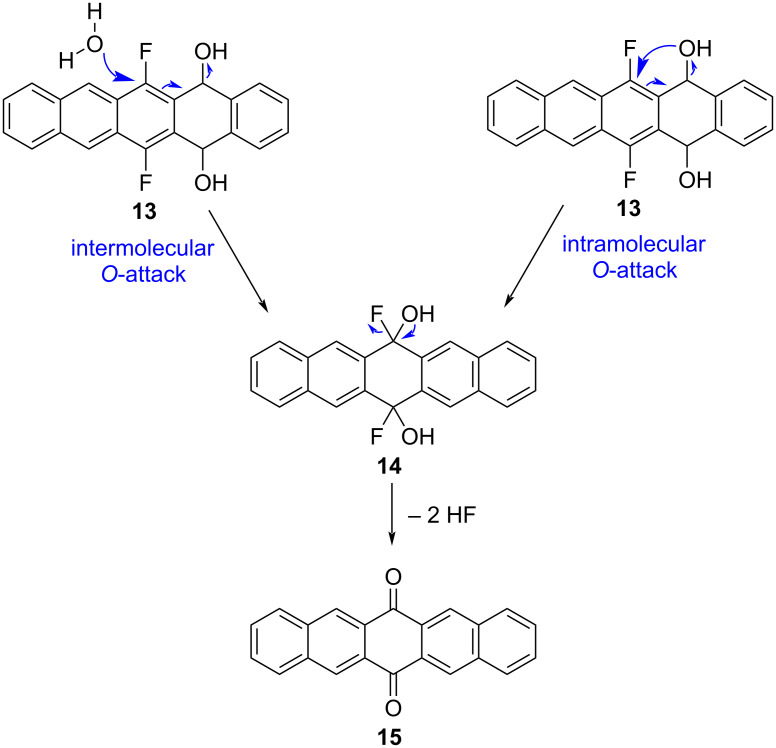
Decomposition of diol **13** in solution.

The low yield for the formation of **13** seems to be an intrinsic instability of its *ortho*-fluorobenzylic alcohol moiety. Moreover, the compound quantitatively decomposes to 6,13-pentacenequinone **15** in CD_2_Cl_2_ solution at room temperature within 2 h. A possible reaction pathway for this degradation in the presence of moisture is an intermolecular attack of water, leading to a fluorohydrine **14** (Scheme 3). In absence of water, this reaction might take place in an intramolecular manner. The fluorohydrine **14** then rapidly decomposes to 6,13-pentacenequinone (**15**) after elimination of HF.

The final aromatization of diol **13** to the target molecule **5** proceeded smoothly in 74% yield using SnCl_2_ in 1,4-dioxane and aqueous HCl [11–12]. F2PEN **5** can be stored under inertgas atmosphere at −20 °C for a month without noticeable decomposition. However, in solution under ambient atmosphere and sunlight decomposition takes place quickly, which is indicated by decolorization of a purple solution in CH_2_Cl_2_ within 3 min. ^1^H NMR analysis showed 6,13-pentacenequinone (**15**) as the degradation product. The rate of degradation in degassed C_6_D_6_ was slow enough to obtain a clean ^1^H NMR spectrum (see Supporing Information File 1) [17].

**Figure 2:**
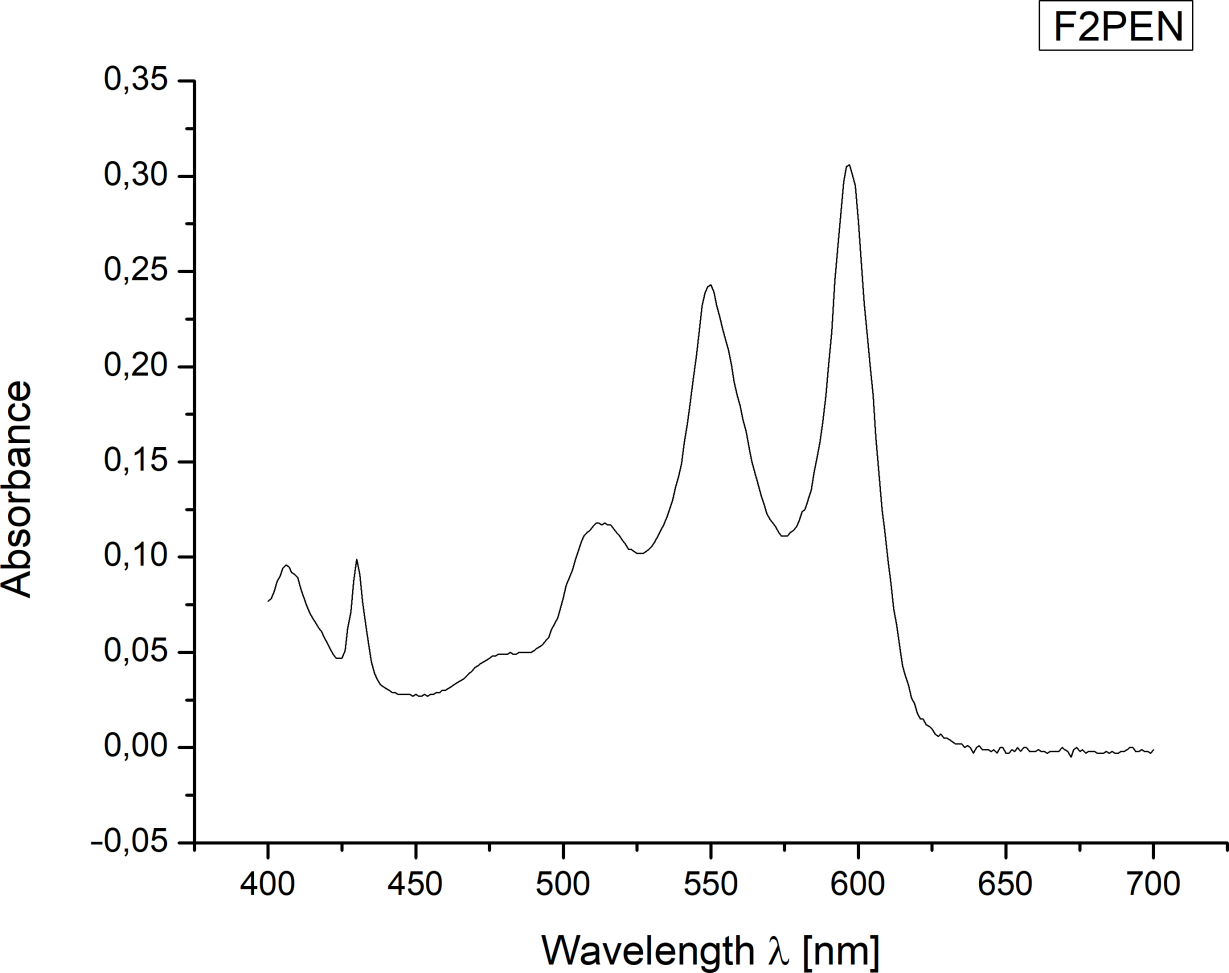
UV–vis spectrum of F2PEN **5** in CH_2_Cl_2_.

Next, the HOMO–LUMO gap of **5** was investigated via UV–vis spectroscopy in solution (Figure 2). The λ_max_ was determined to be at 597 nm, which corresponds to a HOMO–LUMO gap of Δ*E* = 2.08 eV. Comparison with the values of PEN (Δ*E* = 2.13 eV) [9] and PFP (Δ*E* = 1.99 eV) [9] shows that the electronic structure of F2PEN **5** lies in between these two. While fluorine substituents in 2,3,9,10-positions (F4PEN **3**) result in a larger HOMO–LUMO gap compared to PEN [7–8], substitution in the 6,13-positions leads to smaller band-gaps, similar to the unilaterally substituted F6PEN **4** [9].

## Conclusion

Starting from 1,4-difluorobenzene a synthetic route to 6,13-difluoropentacene was established (7% over seven steps). Key steps are Friedel–Crafts acylation reactions and an *ortho*-directed lithiation of 1,4-difluoroanthracene. This strategy could be applicable for the synthesis of differently substituted 6,13-difluoropentacenes as well.

## Supporting Information

File 1 Experimental details, spectroscopic and analytical data of all new compounds.

## References

[1] Ruiz R, Choudhary D, Nickel B, Toccoli T, Chang K-C, Mayer A C, Clancy P, Blakely J M, Headrick R L, Iannotta S (2004). Chem Mater.

[2] Anthony J E (2008). Angew Chem, Int Ed.

[3] Bunz U H F (2015). Acc Chem Res.

[4] Hains A W, Liang Z, Woodhouse M A, Gregg B A (2010). Chem Rev.

[5] Tang M L, Bao Z (2011). Chem Mater.

[6] Sakamoto Y, Suzuki T, Kobayashi M, Gao Y, Fukai Y, Inoue Y, Sato F, Tokito S (2004). J Am Chem Soc.

[7] Shen B, Geiger T, Einholz R, Reicherter F, Schundelmeier S, Maichle-Mössmer C, Speiser B, Bettinger H F (2018). J Org Chem.

[8] Geiger T, Schundelmeier S, Hummel T, Ströbele M, Leis W, Seitz M, Zeiser C, Moretti L, Maiuri M, Cerullo G (2020). Chem – Eur J.

[9] Hofmann P E, Tripp M W, Bischof D, Grell Y, Schiller A L C, Breuer T, Ivlev S I, Witte G, Koert U (2020). Angew Chem, Int Ed.

[10] Tykwinski R R (2019). Acc Chem Res.

[11] Schwaben J, Münster N, Klues M, Breuer T, Hofmann P, Harms K, Witte G, Koert U (2015). Chem – Eur J.

[12] Schwaben J, Münster N, Breuer T, Klues M, Harms K, Witte G, Koert U (2013). Eur J Org Chem.

[R13] 13Preparations of alkyl-disubstituted 6,13-difluoropentacenes via the corresponding pentacenequinone are reported in a patent: Okamoto, K; *Jpn. Kokai Tokkyo Koho*, JP 2008024684, 07.02.2008.

[14] Krapcho A P, Getahun Z (1985). Synth Commun.

[15] Wang J, Leung L M (2013). Dyes Pigm.

[16] Parham W E, Piccirilli R M (1976). J Org Chem.

[R17] 17Because of the low solubility and decomposition in solution, no ^13^C NMR spectrum could be obtained.

