# Supplementary material for: Synthesis of 6,13-difluoropentacene
Source: Beilstein J Org Chem. 2020 Sep 2;16:2136–40. doi: 10.3762/bjoc.16.181 (PMC7476596; doi:10.3762/bjoc.16.181)
Supplement: File 1 — Experimental details, spectroscopic and analytical data of all new compounds. [file Beilstein_J_Org_Chem-16-2136-s001.pdf]

BEILSTEIN JOURNAL OF ORGANIC CHEMISTRY

Supporting Information

for

Synthesis of 6,13-difluoropentacene

Matthias W. Tripp and Ulrich Koert

Beilstein J. Org. Chem. **2020,** *16,* 2136–2140. doi:10.3762/bjoc.16.181

Experimental details, spectroscopic and analytical data of all new compounds

License and Terms: This is a supporting information file under the terms of the Creative Commons Attribution License (https://creativecommons.org/licenses/by/4.0). Please note that the reuse, redistribution and reproduction in particular requires that the authors and source are credited.
The license is subject to the *Beilstein Journal of Organic Chemistry* terms and conditions: (https://www.beilstein-journals.org/bjoc)

Table of contents

1. Methods and materials S3
2. Synthesis of 6,13-difluoropentacene S5
3. NMR spectra of all compounds S12
4. References S19

1. Methods and materials

General information

All anhydrous reactions were carried out using flame-dried glassware under argon atmosphere. All solvents were distilled by rotary evaporation. THF for anhydrous reactions was dried with KOH and subsequently distilled from sodium/benzophenone and from Solvona® respectively. All other solvents employed under anhydrous and/or anaerobic conditions were bought in anhydrous form. C_6D_6 for NMR-measurements was degassed three times by freeze-pump-thaw cycles prior to use. All commercially available reagents and reactants were used without further purification unless otherwise noted. Reactions were monitored by thin layer chromatography (TLC) using Merck Silica Gel 60 F_{254} and visualized by fluorescence quenching under UV-light. In addition, TLC-plates were stained using a cerium sulfate/phosphomolybdic acid stain or a potassium permanganate stain. Chromatographic purification of products was performed on Macherey-Nagel Silica Gel 60 (230–400 mesh) using a forced flow of eluents. All crude products were adsorbed onto silica by dissolving in an appropriate solvent and removing the solvent under reduced pressure. Concentration under reduced pressure was performed by rotary evaporation at 40 °C and appropriate pressure and by exposing to high vacuum at room temperature if necessary.

NMR-Spectroscopy

NMR spectra were recorded on a Bruker AVIII HD250, AVII 300, AVIII HD300, AVIII 500 or AVIII HD500 spectrometer at room temperature unless otherwise mentioned. Chemical shifts are reported in ppm with the solvent resonance as internal standard. All reported ^{19}F-NMR spectra are proton decoupled ^{19}F{^{1}H}-measurements and referenced to external $CFCl_3$. Data are reported as follows: s = singlet, d = doublet, t = triplet, q = quartet, quin = quintet, m = multiplet and combination thereof. Due to the low solubility of compounds **5** & **16** no ^{13}C NMR spectra were obtained. All correlations of atoms from NMR spectra of new compounds could be achieved via additional 2D-NMR data (HSQC- and HMBC-spectra) which is not shown within this Supporting Information.

High resolution mass spectrometry

HR-ESI and APCI mass spectra were acquired with a Finnigan LTQ-FT Ultra mass spectrometer (Thermo Fischer Scientific). EI mass spectra were acquired with an AccuTOF GCv (Jeol) mass spectrometer.

Infrared spectroscopy

FTIR spectra were recorded on a BRUKER IFS 200 spectrometer. Intensities are reported as follows: s = strong, m = medium, w = weak.

Melting points

Melting points were determined on a MP70 (Mettler Toledo) using one end closed capillary tubes.

UV–vis spectroscopy

UV–vis-spectra have been acquired with a Thermo Scientific Multiskan Go using quartz rectangular cuvettes (Teflon cover, 10 mm light path, 3.5 mL) by Aldrich.

2. Synthesis of 6,13-difluoropentacene

1,4-Difluoroanthracene-9,10-dione (11)[1]

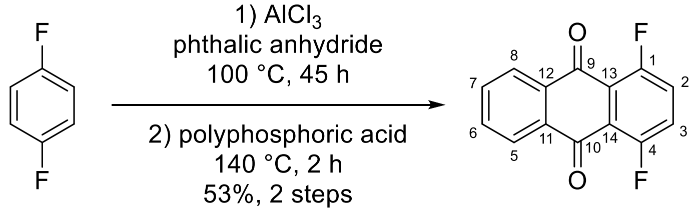

10 [114.09] **11** [244.20]

Following an adapted procedure of Krapcho et al., a suspension of phthalic anhydride (4.30 g, 29.0 mmol, 1.00 equiv) and $AlCl_3$ (15.4 g, 116 mmol, 4.00 equiv) in 1,4-difluorobenzene **10** (30 mL, 290 mmol, 10.0 equiv) was stirred at 100 °C for 45 h. Excess 1,4-difluorobenzene (26 mL, 253 mmol, 8.72 eq.) was reisolated *via* distillation and the residue was taken up in $CHCl_3$ (250 mL) and 1 M a. HCl (170 mL). The layers were separated and the aqueous layer extracted with $CHCl_3$ (3 × 80 mL). The combined organic layers were dried over $MgSO_4$ and the solvent was removed under reduced pressure. The residue was dissolved in $CHCl_3$ (20 mL) and *n*-hexane (45 mL) was added. The mixture was incubated at −20 °C for 16 h and the precipitate was filtered and dried under reduced pressure.

The crude 2-(2,5-difluorobenzoyl)benzoic acid was suspended in polyphosphoric acid (41.2 g) and the suspension was stirred at 140 °C for 2 h. The mixture was cooled to room temperature and poured into ice-water (200 mL) and CH_2Cl_2 (200 mL) and neutralized to pH = 7 using K_2CO_3. The layers were separated and the aqueous layer was extracted with CH_2Cl_2 (3 × 150 mL). The combined organic layers were dried over $MgSO_4$ and filtered over Alox (basic). The crude product was adsorbed onto silica and purified via column chromatography (*n*-hexane/$CHCl_3$ 3:7) to obtain anthraquinone **11** (3.72 g, 15.2 mmol, 53% over two steps) as yellow solid.

TLC: R_f = 0.44 (*n*-pentane/EtOAc 5:1).

^{1}H-NMR: 250 MHz, $CDCl_3$; δ = 8.26 (dd, J = 5.8, 3.3 Hz, 2H, *H6* & *H7*), 7.81 (dd, J = 5.8, 3.3 Hz, 2H, *H5* & *H8*), 7.51-7.46 (m, 2H, *H2* & *H3*) ppm.

^{13}C-NMR: 75 MHz, $CDCl_3$; δ = 180.7 (s, 2C, *C9* & *C10*), 157.8 (dd, J = 268.5, 4.0 Hz, 2C, *C1* & *C4*), 134.5 (s, 2C, *C5* & *C8*), 133.4 (s, 2C, *C11* &

C12), 127.1 (s, 2C, *C6* & *C7*), 124.7 (dd, J = 19.5, 15.1 Hz, 2C, *C2* & *C3*), 121.8 (d, J = 2.2 Hz, 2C, *C13* & *C14*) ppm.

^{19}F-NMR: 235 MHz, $CDCl_3$; δ = –114.3 (s, 2F, *F1* & *F4*) ppm.

HR-MS: EI(+); *m/z* calc. for $C_{14}H_6F_2O_2$ $[M]^+$: 244.03359, found: 244.03415.

FT-IR: film; $\tilde{\nu}$ = 3087 (w), 2926 (w), 1777 (w), 1715 (w), 1679 (s), 1591 (m), 1497 (w), 1469 (w), 1416 (m), 1337 (m), 1311 (w), 1287 (w), 1253 (s), 1170 (w), 1082 (w), 1064 (w), 1024 (w), 935 (w), 892 (w), 870 (w), 843 (w), 819 (w), 798 (w), 746 (w), 722 (m), 690 (w), 653 (w), 565 (w), 421 (w) cm^{-1}.

m.p.: 232 °C ($CDCl_3$).

1,4-Difluoroanthracene (9)

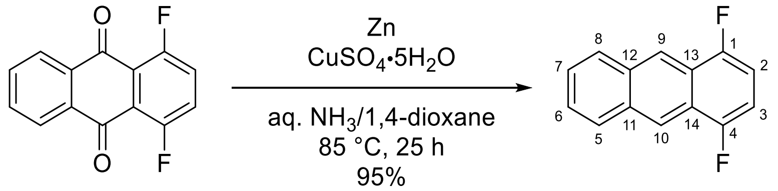

11 [244.20] **9** [214.21]

A suspension of anthraquinone **11** (200 mg, 819 µmol, 1.00 equiv), zinc powder (214 mg, 3.28 mmol, 4.00 equiv) and $CuSO_4 \cdot 5H_2O$ (10 mg, 41.0 µmol, 0.05 equiv) in 1,4-dioxane (4.0 mL) and aq NH_3 (25%, 4.0 mL) was stirred at 85 °C for 25 h in a pressure tube under vigorous stirring. The suspension was filtered over celite and washed with 2 M aq HCl (60 mL). The aqueous layer was reextracted with EtOAc (2 × 60 mL) and the combined organic layers were washed with brine (30 mL) and dried over $MgSO_4$. The crude product was adsorbed onto silica and purified via column chromatography (*n*-pentane) to obtain anthracene **9** (166 mg, 775 µmol, 95%) as colorless solid.

TLC: R_f = 0.81 (*n*-pentane/EtOAc 50:1).

^{1}H-NMR: 500 MHz, $CDCl_3$; δ = 8.65 (s, 2H, *H9* & *H10*), 8.06 (dd, J = 6.4, 3.3 Hz, 2H, *H5* & *H8*), 7.55 (dd, J = 6.6, 3.2 Hz, 2H, *H6* & *H7*), 7.00 (dd, J = 7.4, 6.7 Hz, 2H, *H2* & *H3*) ppm.

^{13}C-NMR: 126 MHz, $CDCl_3$; δ = 154.9 (dd, J = 252.4, 6.0 Hz, 2C, *C1* & *C4*), 132.1 (s, 2C, *C11* & *C12*), 128.7 (s, 2C, *C5* & *C8*), 126.8 (s, 2C, *C6* & *C7*), 123.3 (dd, J = 14.5, 11.0 Hz, 2C, *C13* & *C14*), 120.1 (t, J = 2.7 Hz, 2C, *C9* & *C10*), 106.5 (dd, J = 19.0, 12.4 Hz, 2C, *C2* & *C3*) ppm.

^{19}F-NMR: 283 MHz, $CDCl_3$; δ = –127.0 (s, 2F, *F1* & *F4*) ppm.

HR-MS: EI(+); *m/z* calc. for $C_{14}H_8F_2$ $[M]^+$: 214.05941, found: 214.05990.

FT-IR: film; $\tilde{\nu}$ = 3058 (w), 2960 (w), 2928 (w), 2855 (w), 2254 (w), 1650 (w), 1581 (w), 1462 (w), 1382 (w), 1320 (w), 1261 (w), 1237 (w), 1202 (w), 1139 (w), 1096 (w), 1019 (w), 957 (w), 904 (s), 818 (w), 725 (s), 650 (w), 612 (w), 468 (w), 434 (w) cm^{-1}.

m.p.: 107 °C ($CDCl_3$).

2-(1,4-Difluoroanthracene-2-carbonyl)benzoic acid (12)

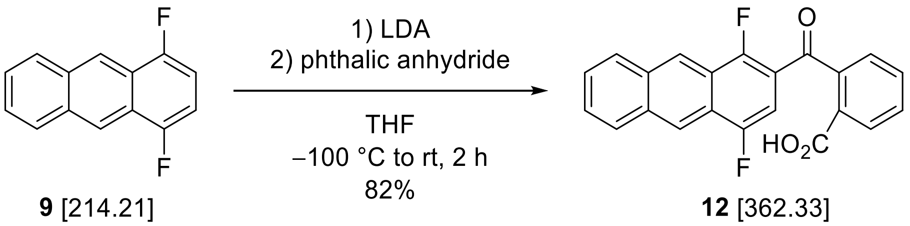

9 [214.21] **12** [362.33]

n-BuLi (2.5 M in *n*-hexane, 196 µL, 490 µmol, 1.05 equiv) was added dropwise to a solution of *N*,*N*-diisopropylamine (72.2 µL, 514 µmol, 1.10 equiv) in THF (1.2 mL) at 0 °C. The mixture was stirred at 0 °C for 15 min and then cooled to −78 °C. A solution of anthracene **9** (100 mg, 467 µmol, 1.00 eq.) in THF (1.0 mL) was added dropwise and the resulting orange solution was stirred at −78 °C for 15 min. The mixture was added to a suspension of phthalic anhydride (83 mg, 560 µmol, 1.20 equiv) in THF (1.8 mL) at −100 °C and stirred at this temperature for 1 h. The cooling bath was removed and the solution was stirred additional 1 h at room temperature before 2 M aq HCl (25 mL) was added. The mixture was extracted with EtOAc (2 × 30 mL) and the combined organic layers were dried over $MgSO_4$. The crude product was adsorbed

onto silica and purified via column chromatography (*n*-pentane/EtOAc/AcOH 5:1:0.1) to obtain carboxylic acid **12** (138 mg, 381 µmol, 82%) as yellow solid.

TLC: R_f = 0.26 (*n*-pentane/EtOAc/AcOH 4:1:0.1).

^{1}H-NMR: 500 MHz, acetone-d_6; δ = 11.64 (s, 1H, COO*H*), 8.76 (s, 1H, $H_{ar.}$), 8.75 (s, 1H, $H_{ar.}$), 8.25 (d, *J* = 8.4 Hz, 1H, $H_{ar.}$), 8.19 (d, *J* = 8.4 Hz, 1H, $H_{ar.}$), 8.15 (d, *J* = 7.5 Hz, 1H, $H_{ar.}$), 7.81 (td, *J* = 7.5, 1.0 Hz, 1H, $H_{ar.}$), 7.74-7.65 (m, 3H, 3 x $H_{ar.}$), 7.59-7.56 (m, 2H, 2 x $H_{ar.}$) ppm.

^{13}C-NMR: 126 MHz, acetone-d_6; δ = 192.7 (s, 1C, *C*O), 167.4 (s, 1C, *C*OOH), 156.5 (d, *J* = 265.0 Hz, 1C, *C*F), 155.4 (dd, *J* = 250.0, 3.0 Hz, 1C, *C*F), 145.6 (s, 1C, $C_{ar.}$), 134.4 (s, 1C, $C_{ar.}$), 133.7 (s, 1C, $C_{ar.}$), 133.3 (d, *J* = 2.0 Hz, 1C, $C_{ar.}$), 131.0 (s, 1C, $C_{ar.}$), 130.5 (s, 1C, $C_{ar.}$), 129.7 (s, 1C, $C_{ar.}$), 129.5 (s, 1C, $C_{ar.}$), 129.4 (s, 1C, $C_{ar.}$), 129.1 (s, 1C, $C_{ar.}$), 128.4 (s, 1C, $C_{ar.}$), 127.6 (s, 1C, $C_{ar.}$), 125.3-125.1 (m, 1C, $C_{ar.}$), 123.7-123.5 (m, 2C, 2 x $C_{ar.}$), 120.8 (s, 1C, $C_{ar.}$), 119.0-118.9 (m, 1C, $C_{ar.}$), 106.2 (d, *J* = 24.0 Hz, 1C, $C_{ar.}$) ppm.

^{19}F-NMR: 235 MHz, acetone-d_6; δ = –119.4 (d, *J* = 22.2 Hz, 1F), –127.7 (d, *J* = 22.0 Hz, 1F) ppm.

HR-MS: ESI(–); *m/z* calc. for $C_{22}H_{11}F_2O_3$ $[M-H]^-$: 361.0682, found: 361.0683.

FT-IR: film; $\tilde{\nu}$ = 3058 (w), 2658 (w), 2542 (w), 1774 (w), 1691 (w), 1665 (w), 1639 (s), 1596 (w), 1577 (w), 1541 (w), 1488 (w), 1438 (w), 1410 (w), 1389 (w), 1369 (s), 1329 (m), 1285 (w), 1265 (w), 1233 (s), 1164 (w), 1142 (m), 1109 (w), 1076 (w), 1056 (w), 974 (w), 885 (m), 829 (w), 771 (w), 736 (s), 712 (w), 667 (m), 644 (w), 559 (w), 471 (m) cm^{-1}.

m.p.: 203 °C decomposition (toluene).

6,13-Difluoropentacene-5,14-dione (16)

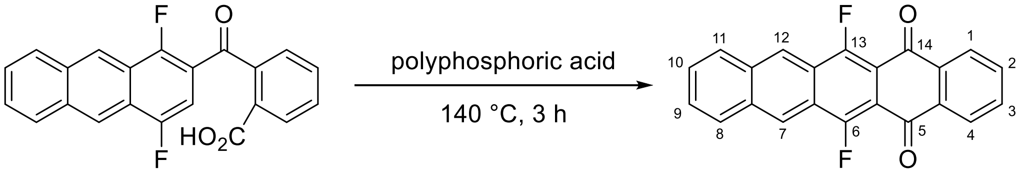

12 [362.33] **16** [344.32]

A suspension of carboxylic acid **12** (30 mg, 82.8 µmol, 1.00 equiv) in polyphosphoric acid (1.30 g) was stirred at 140 °C for 3 h. The mixture was cooled to room temperature and taken up in CH_2Cl_2 (30 mL) and H_2O (30 mL). The layers were separated and the aqueous layer was extracted with CH_2Cl_2 (2 × 30 mL). The combined organic layers were dried over $MgSO_4$ and the solvent was removed under reduced pressure to obtain the crude pentacenequinone **16** as red solid, which was used in the next step without further purification.

TLC: R_f = 0.49 (CH_2Cl_2).

^{1}H-NMR: 300 MHz, CD_2Cl_2; δ = 8.98 (s, 2H, *H7* & *H12*), 8.33 (dd, *J* = 5.8, 3.3 Hz, 2H, 2 x $H_{ar.}$), 8.20 (dd, *J* = 6.4, 3.3 Hz, 2H, 2 x $H_{ar.}$), 7.84 (dd, *J* = 5.8, 3.3 Hz, 2H, 2 x $H_{ar.}$), 7.73 (dd, *J* = 6.5, 3.2 Hz, 2H, 2 x $H_{ar.}$) ppm.

^{19}F-NMR: 235 MHz, CD_2Cl_2; δ = –117.9 (s, 2F, *F6* & *F13*) ppm.

HR-MS: EI(+); *m/z* calc. for $C_{22}H_{10}F_2O_2$ $[M]^+$: 344.06489, found: 344.06531.

FT-IR: film; $\tilde{\nu}$ = 2957 (w), 2920 (s), 2851 (w), 1775 (w), 1744 (w), 1678 (m), 1587 (w), 1530 (w), 1446 (m), 1384 (w), 1364 (w), 1344 (w), 1284 (w), 1256 (s), 1152 (w), 1092 (w), 1011 (s), 978 (w), 886 (w), 800 (s), 748 (w), 725 (m), 532 (w), 467 (w) cm^{-1}.

m.p.: 238 °C decomposition (CH_2Cl_2).

6,13-Difluoro-5,14-dihydropentacene-5,14-diol (13)

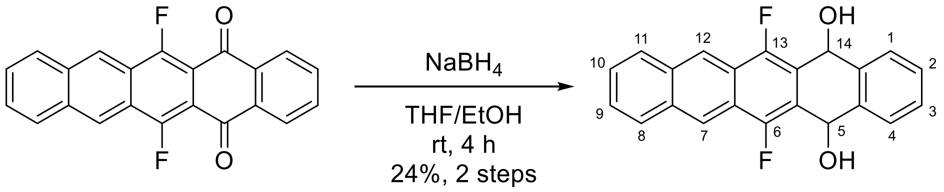

16 [344.32] **13** [348.35]

A suspension of pentacenequinone **16** (crude, ≈82.8 µmol, 1.00 equiv) and $NaBH_4$ (16 mg, 414 µmol, 5.00 equiv) in THF (3.1 mL) and EtOH (2.0 mL) was stirred at room temperature under exclusion of light. After 2.5 h, additional $NaBH_4$ (16 mg, 414 µmol, 5.00 equiv) was added and the mixture was stirred for another 1.5 h. The reaction was cooled to 0 °C and quenched with silica (1.00 g) until gas evolution stopped. The crude product was adsorbed onto the silica and purified via column chromatography (*n*-pentane/Et_2O 3:2 → 0:1) to obtain diol **13** (7 mg, 20.1 µmol, 24% over two steps) as yellow solid.

TLC: R_f = 0.31 (CH_2Cl_2/EtOAc 20:1).

^{1}H-NMR: 300 MHz, CD_2Cl_2; δ = 8.72 (s, 2H, *H7* & *H12*), 8.11 (dd, *J* = 6.5, 3.4 Hz, 2H, 2 x $H_{ar.}$), 7.78 (dd, *J* = 5.7, 3.4 Hz, 2H, 2 x $H_{ar.}$), 7.59 (dd, *J* = 6.6, 3.2 Hz, 2H, 2 x $H_{ar.}$), 7.48 (dd, *J* = 5.7, 3.3 Hz, 2H, 2 x $H_{ar.}$), 6.37 (d, *J* = 2.3 Hz, 2H, *H5* & *H14*), 2.88 (s, 2H, 2 x O*H*) ppm.

^{19}F-NMR: 235 MHz, CD_2Cl_2; δ = –131.1 (s, 2F, *F6* & *F13*) ppm.

HR-MS: APCI(–); *m/z* calc. for $C_{22}H_{11}F_2O$ [M-H_2O-H]$^-$: 329.0783, found: 329.0794.

FT-IR: film; $\tilde{\nu}$ = 3315 (s), 3108 (w), 2976 (w), 2927 (w), 2721 (w), 1681 (w), 1653 (w), 1612 (w), 1563 (w), 1477 (m), 1388 (m), 1368 (s), 1341 (w), 1317 (w), 1236 (w), 1175 (w), 1139 (w), 1027 (m), 991 (s), 972 (w), 935 (w), 900 (m), 809 (w), 781 (w), 759 (s), 741 (w), 726 (w), 664 (w), 635 (w), 552 (w), 530 (w), 480 (w), 460 (w) cm^{-1}.

m.p.: 82 °C decomposition (Et_2O).

6,13-Difluoropentacene (F2PEN, 5)

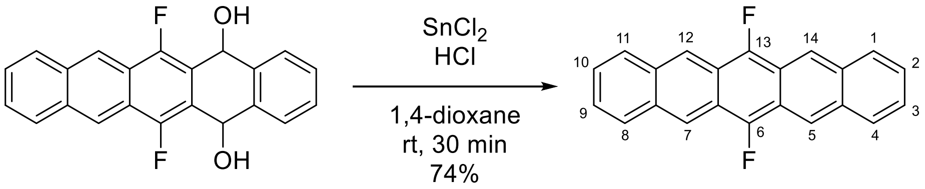

13 [348.35]　　**5** [314.33]

2 M aq HCl (0.20 mL) was added dropwise to a suspension of diol **13** (6 mg, 17.2 µmol, 1.00 equiv) and $SnCl_2$ (33 mg, 172 µmol, 10.0 equiv) in degassed 1,4-dioxane (1.6 mL). The mixture was stirred vigorously for 30 min at room temperature under exclusion of light. The precipitate was centrifuged (13.000 rpm, 3 min) and washed with H_2O (1.0 mL) and acetone (1.0 mL) to obtain F2PEN **5** (4 mg, 12.7 µmol, 74%) as dark purple solid.

^{1}H-NMR: 300 MHz, C_6D_6; δ = 8.82 (s, 4H, *H5* & *H7* & *H12* & *H14*), 7.63 (dd, J = 6.6, 3.2 Hz, 4H, *H1* & *H4* & *H8* & *H11*), 7.04 (dd, J = 6.7, 3.2 Hz, 4H, *H2* & *H3* & *H9* & *H10*) ppm.

^{19}F-NMR: 235 MHz, C_6D_6; δ = –132.1 (s, 2F, *F6* & *F13*) ppm.

HR-MS: EI(+); *m/z* calc. for $C_{22}H_{12}F_2$ $[M]^+$: 314.09071, found: 314.09089.

FT-IR: neat; $\tilde{\nu}$ = 3042 (w), 2960 (w), 2919 (w), 1740 (w), 1670 (w), 1612 (w), 1572 (w), 1485 (w), 1447 (m), 1358 (s), 1341 (w), 1293 (w), 1270 (w), 1205 (w), 1180 (w), 1133 (w), 1050 (w), 1003 (m), 952 (w), 925 (w), 865 (s), 762 (w), 734 (s), 671 (m), 629 (w), 565 (w), 536 (w), 513 (w), 482 (w), 455 (s) cm^{-1}.

m.p.: 288 °C decomposition (1,4-dioxane).

3. NMR spectra of all compounds

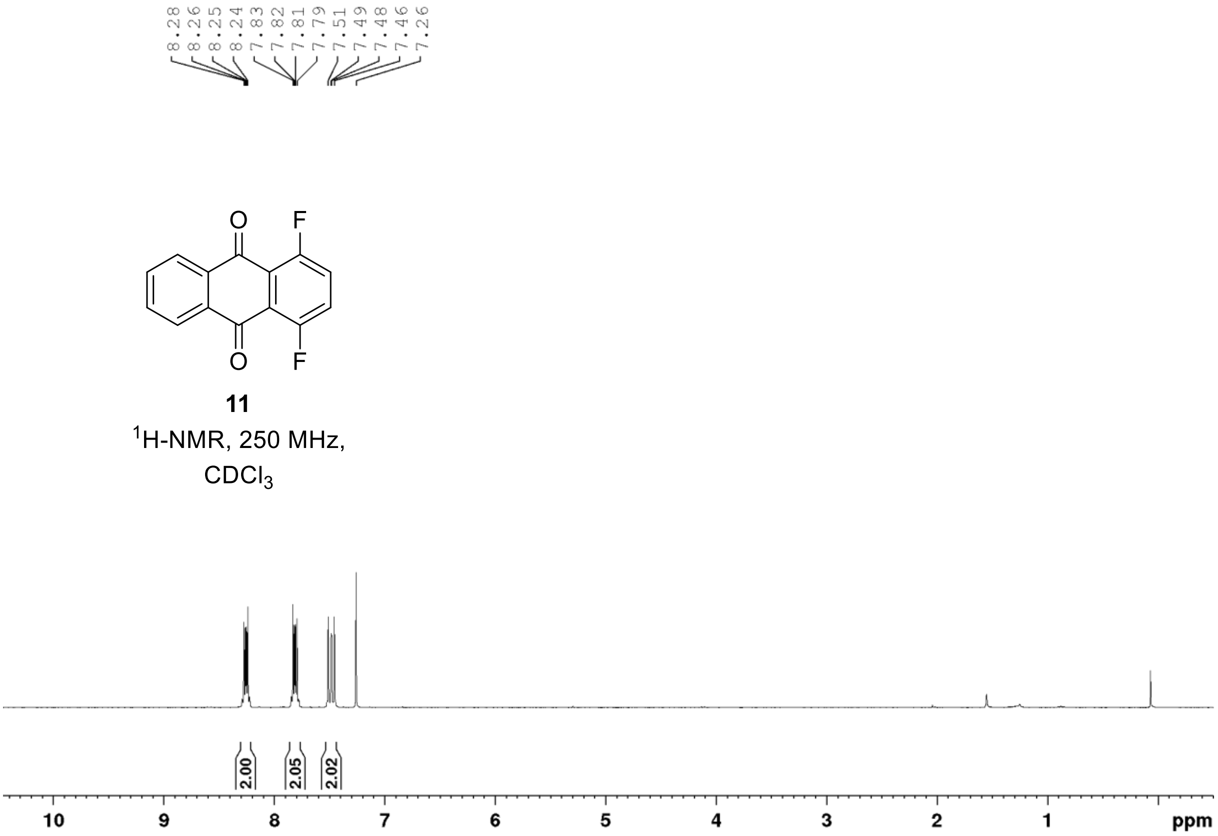

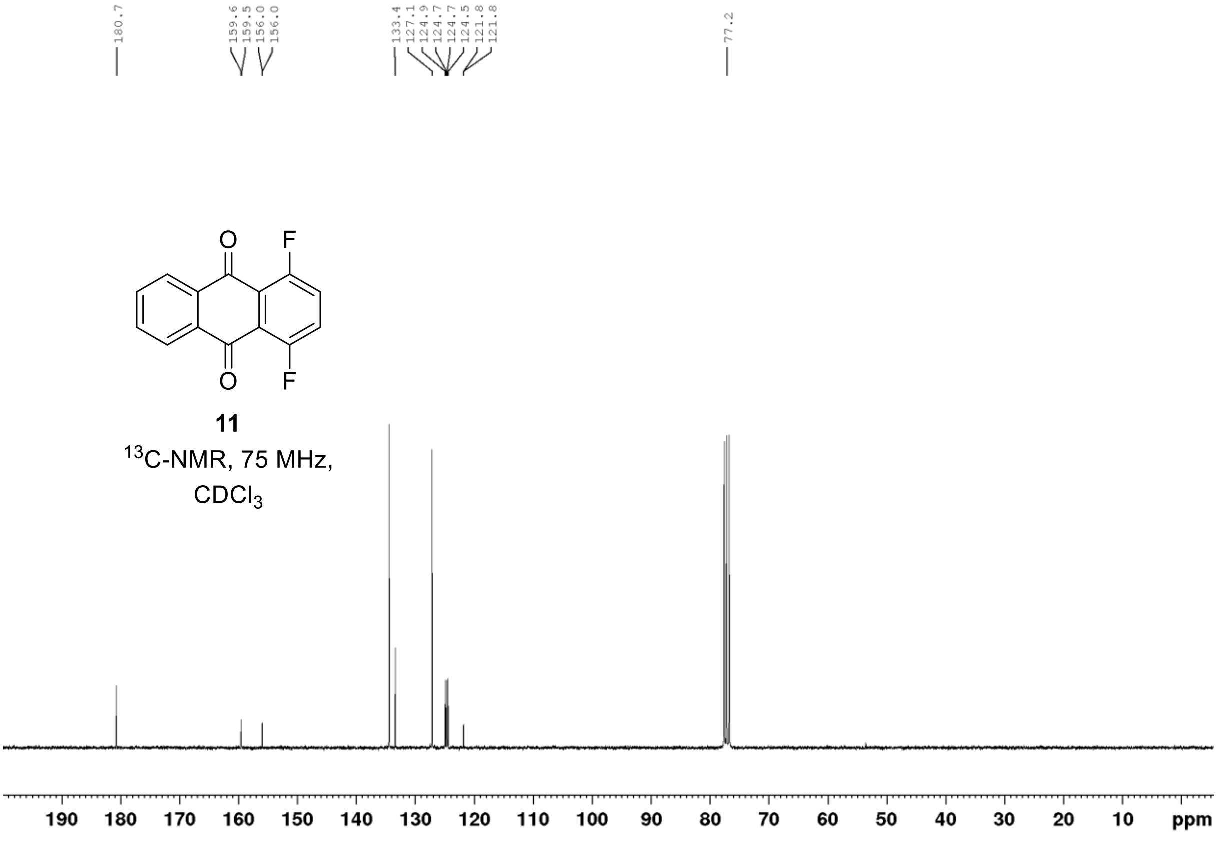

11

^{19}F-NMR, 235 MHz,

$CDCl_3$

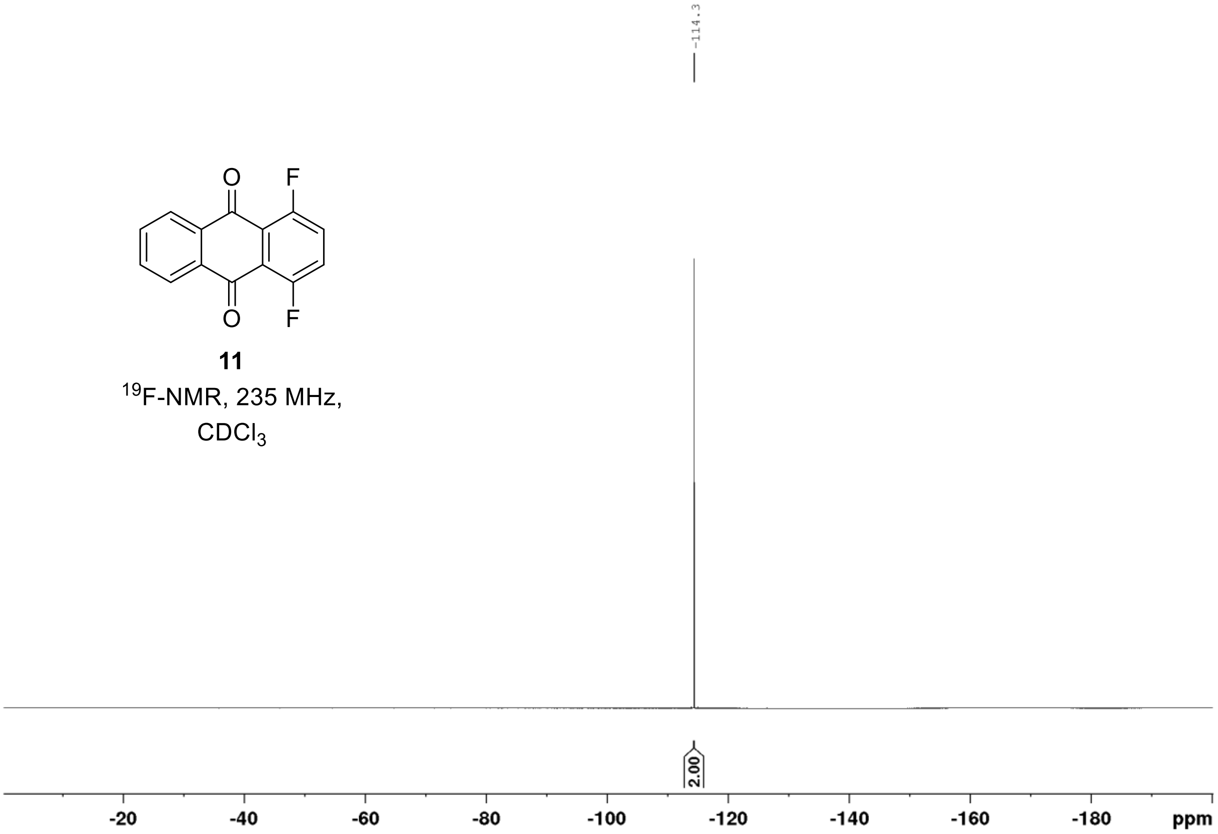

9

^{1}H-NMR, 500 MHz,

$CDCl_3$

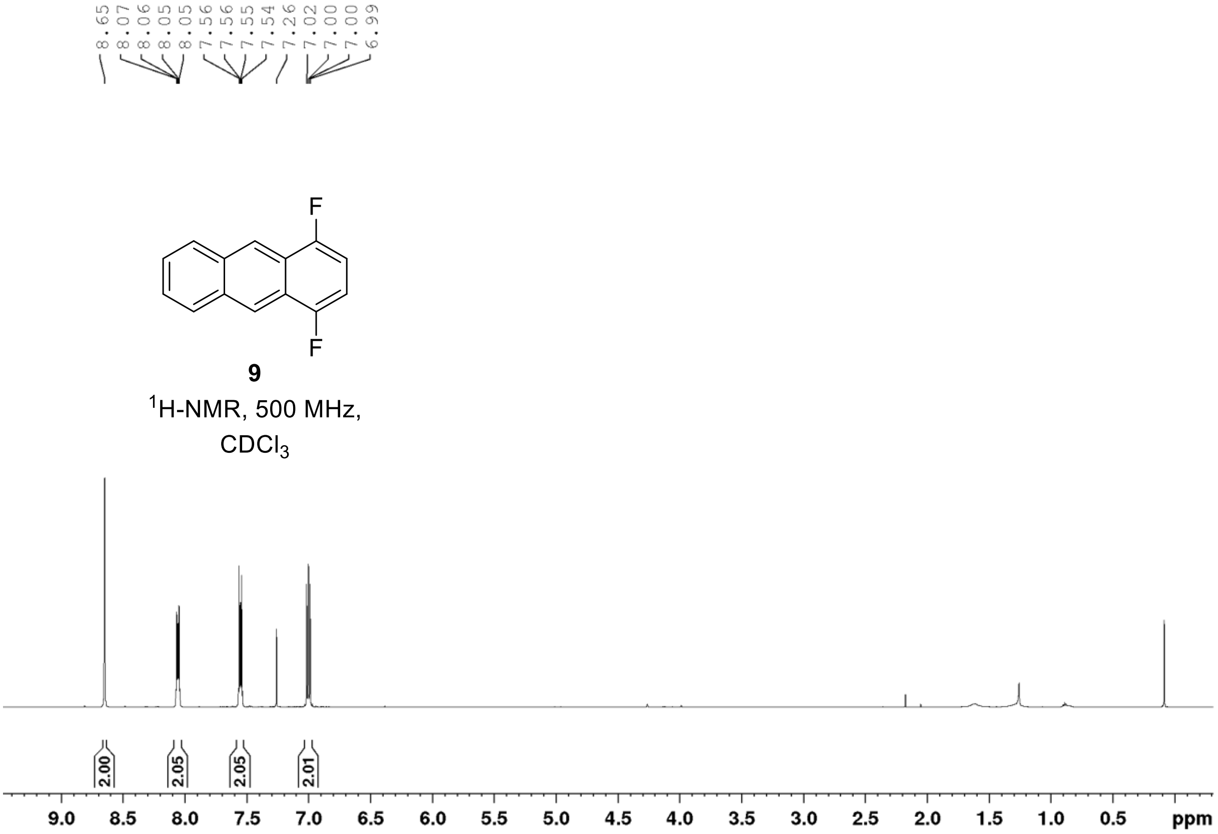

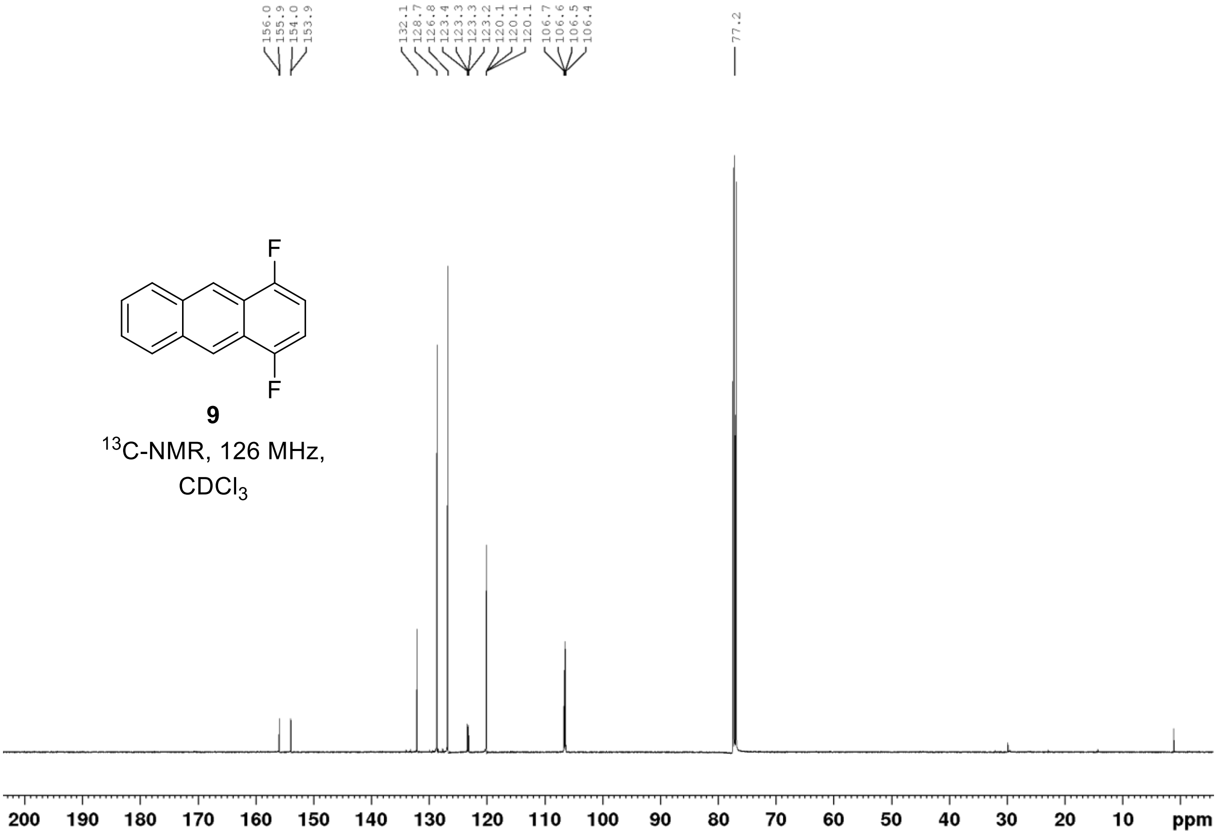
156.0
155.9
154.0
153.9
132.1
128.7
126.8
123.4
123.3
123.3
123.2
120.1
120.1
120.1
106.7
106.6
106.5
106.4
77.2
F
F
9
13C-NMR, 126 MHz,
CDCl3
200 190 180 170 160 150 140 130 120 110 100 90 80 70 60 50 40 30 20 10 ppm

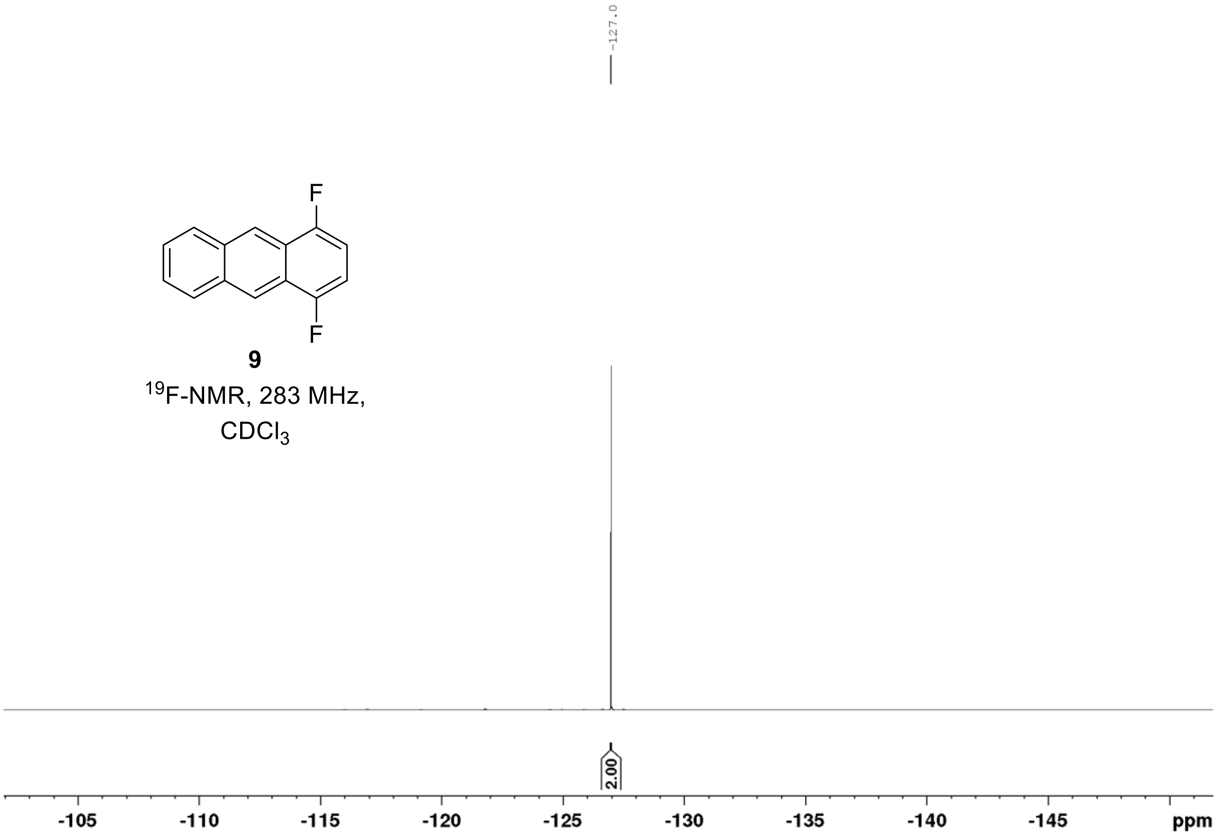
-127.0
F
F
9
19F-NMR, 283 MHz,
CDCl3
2.00
-105 -110 -115 -120 -125 -130 -135 -140 -145 ppm

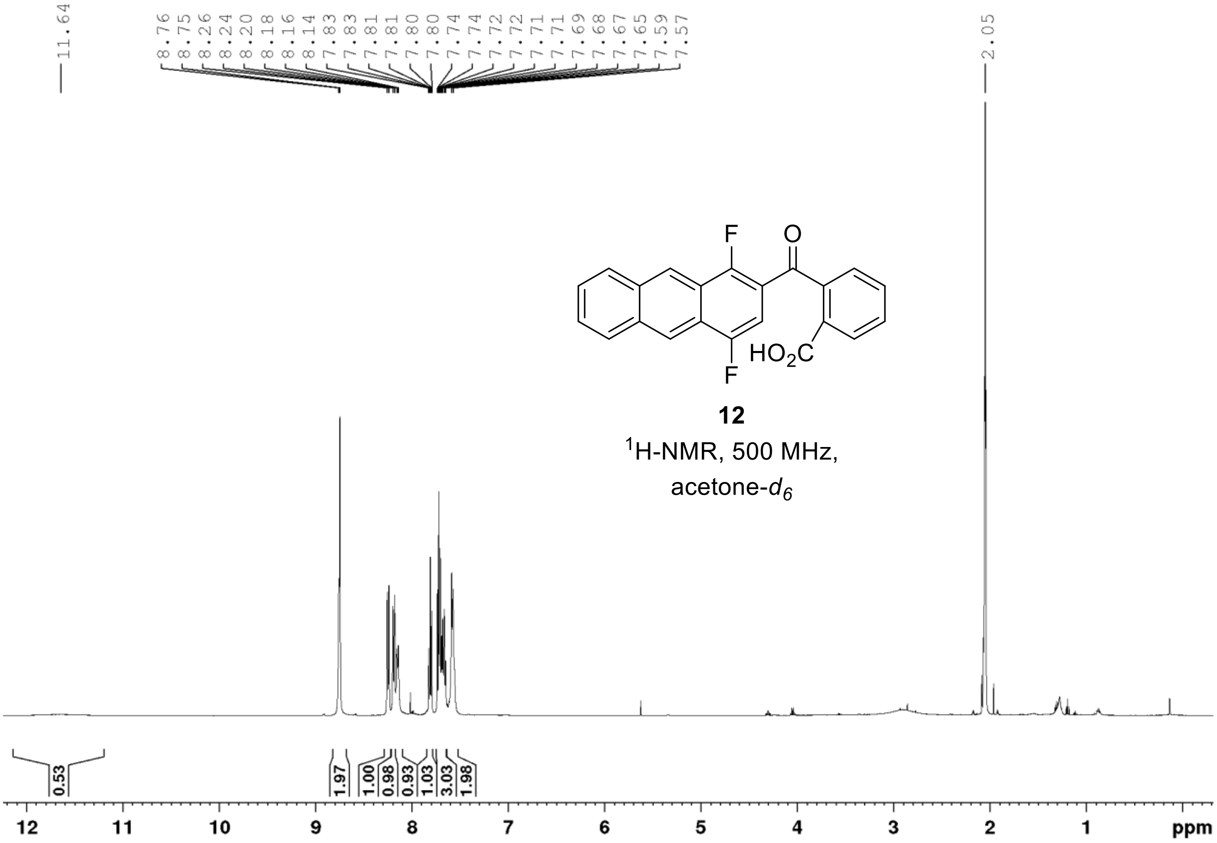
11.64
8.76 8.75 8.26 8.24 8.20 8.18 8.16 8.14 7.83 7.83 7.81 7.81 7.80 7.80 7.74 7.74 7.72 7.72 7.71 7.71 7.69 7.68 7.67 7.65 7.59 7.57
2.05
F
O
HO2C
F
12
1H-NMR, 500 MHz,
acetone-d6
0.53 1.97 1.00 0.98 0.93 1.03 3.03 1.98
12 11 10 9 8 7 6 5 4 3 2 1 ppm

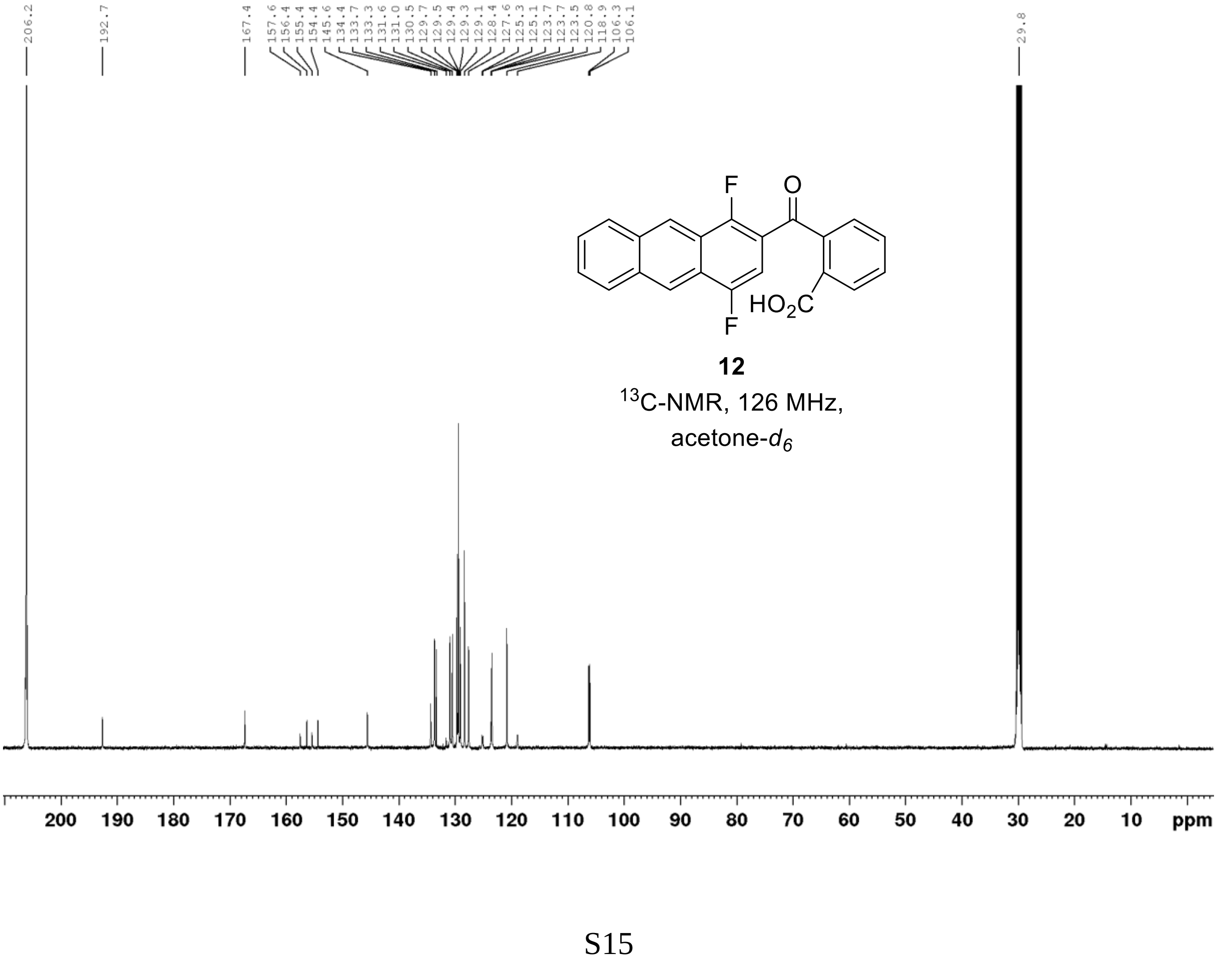
206.2
192.7
167.4
157.6 156.4 155.4 154.4 145.6 134.4 133.7 133.3 131.6 131.0 130.5 129.7 129.5 129.4 129.3 129.1 128.4 127.6 125.3 125.1 123.7 123.5 120.8 118.9 106.3 106.1
29.8
F
O
HO2C
F
12
13C-NMR, 126 MHz,
acetone-d6
200 190 180 170 160 150 140 130 120 110 100 90 80 70 60 50 40 30 20 10 ppm

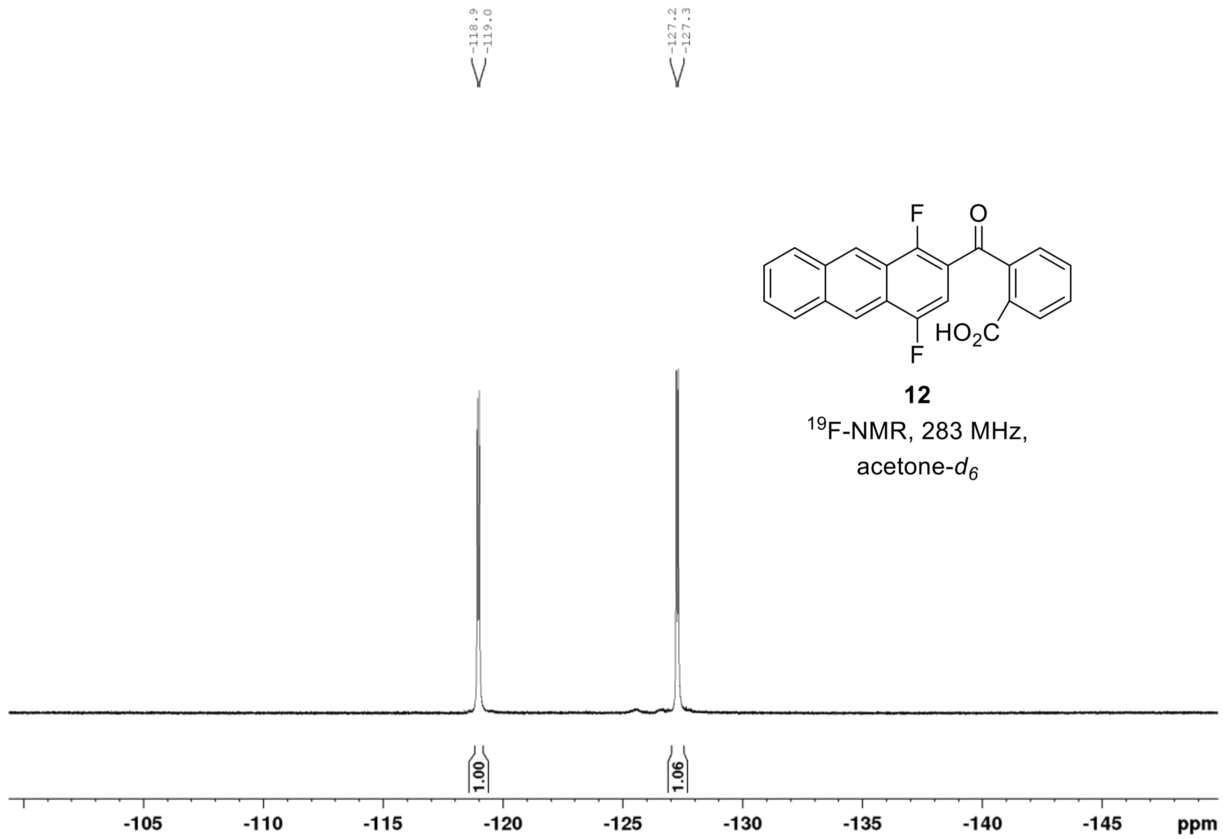
-118.9
-119.0
-127.2
-127.3
F
O
HO2C
F
12
19F-NMR, 283 MHz,
acetone-d6
1.00
1.06
-105 -110 -115 -120 -125 -130 -135 -140 -145 ppm

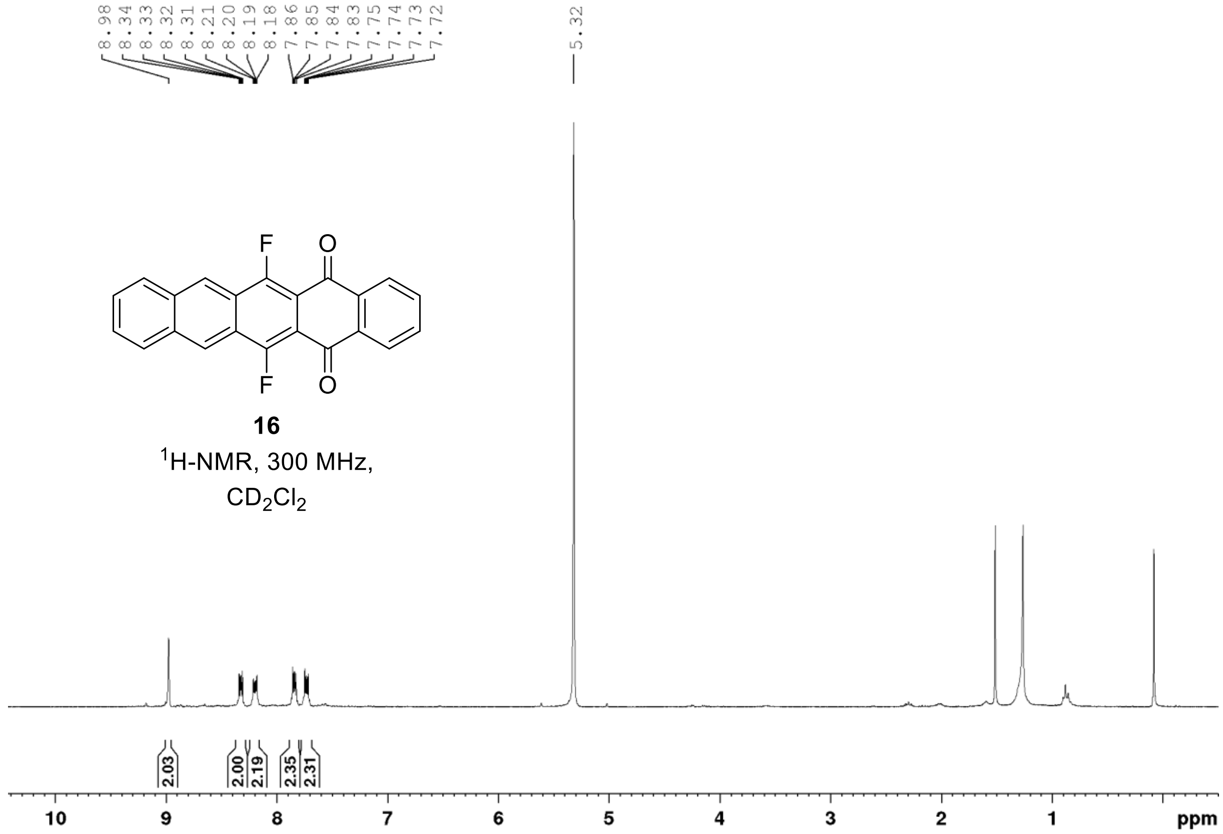
8.98
8.34
8.33
8.32
8.31
8.21
8.20
8.19
8.18
7.86
7.85
7.84
7.83
7.75
7.74
7.73
7.72
5.32
F
O
F
O
16
1H-NMR, 300 MHz,
CD2Cl2
2.03
2.00
2.19
2.35
2.31
10 9 8 7 6 5 4 3 2 1 ppm

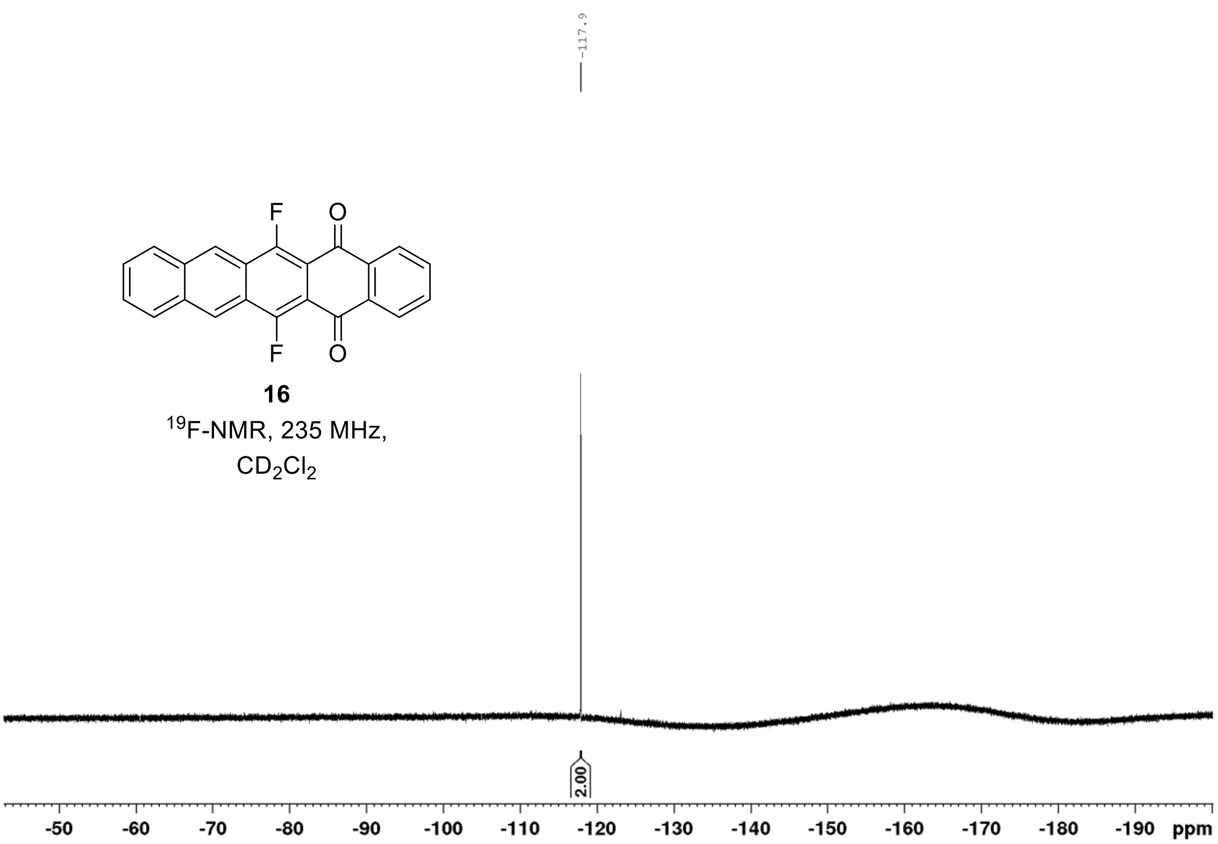

-117.9
F
O
F
O
16
^{19}F-NMR, 235 MHz,
CD_2Cl_2
2.00
-50 -60 -70 -80 -90 -100 -110 -120 -130 -140 -150 -160 -170 -180 -190 ppm

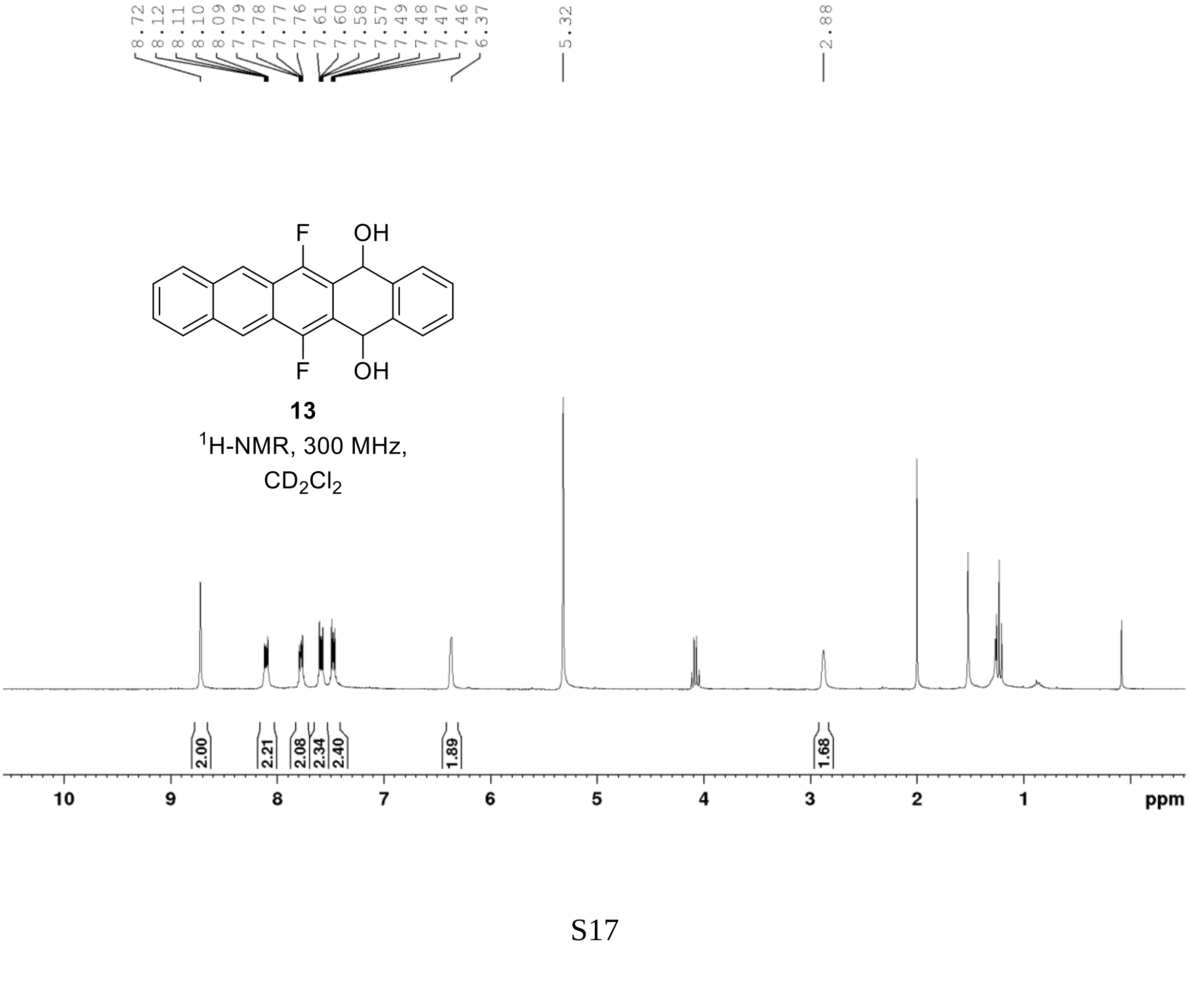

8.72 8.12 8.11 8.10 8.09 7.79 7.78 7.77 7.76 7.61 7.60 7.58 7.57 7.49 7.48 7.47 7.46 6.37
5.32
2.88
F
OH
F
OH
13
^{1}H-NMR, 300 MHz,
CD_2Cl_2
2.00 2.21 2.08 2.34 2.40 1.89 1.68
10 9 8 7 6 5 4 3 2 1 ppm

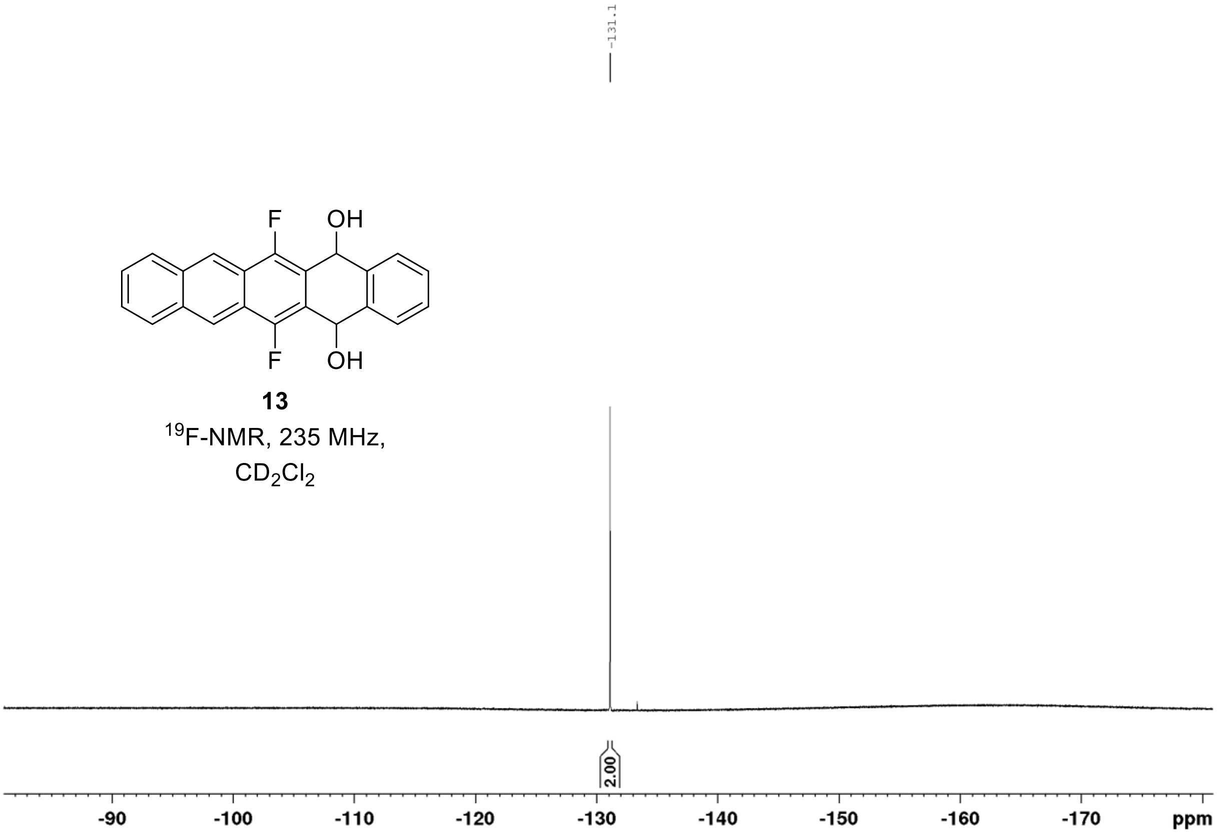
-131.1
F
OH
F
OH
13
19F-NMR, 235 MHz,
CD2Cl2
2.00
-90
-100
-110
-120
-130
-140
-150
-160
-170
ppm

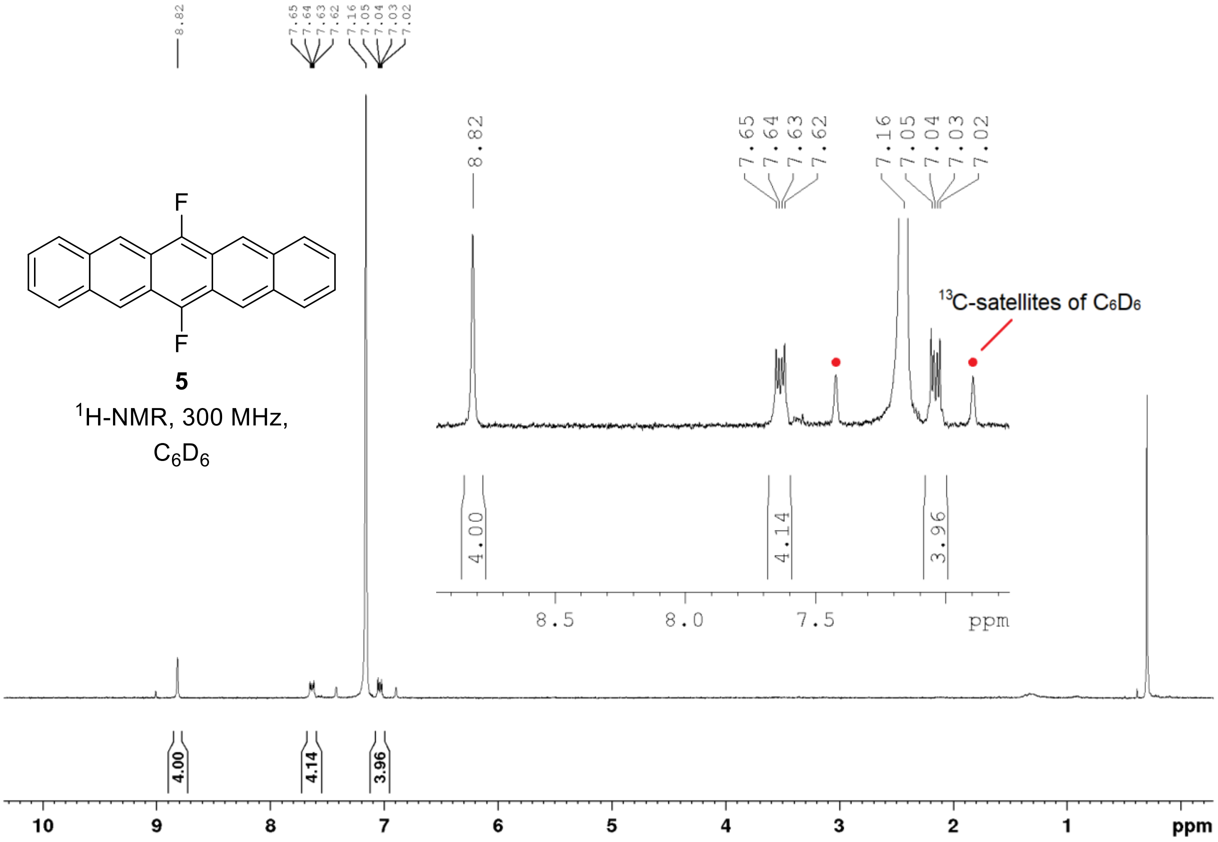
8.82
7.65
7.64
7.63
7.62
7.16
7.05
7.04
7.03
7.02
F
F
5
1H-NMR, 300 MHz,
C6D6
13C-satellites of C6D6
4.00
4.14
3.96
8.5
8.0
7.5
ppm
10
9
8
7
6
5
4
3
2
1
ppm

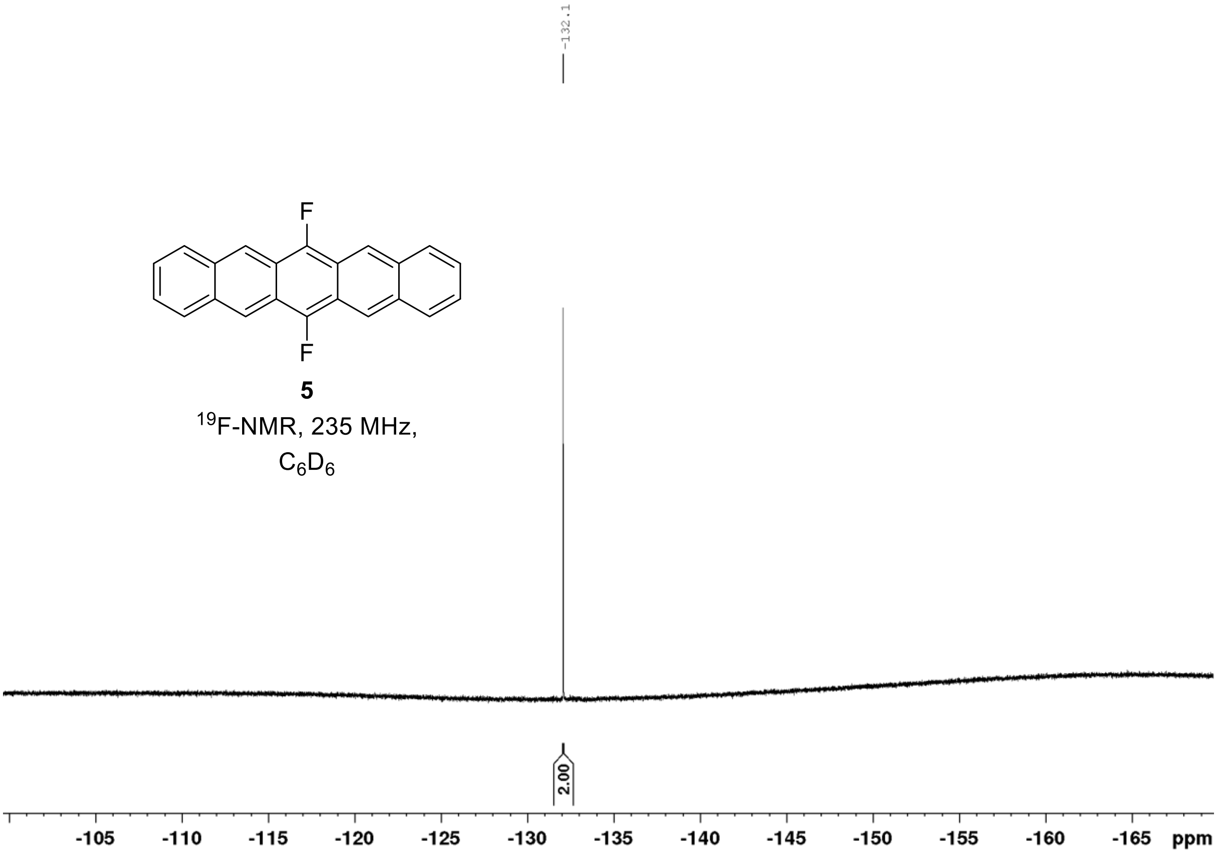

4. References

1. Krapcho, A. P.; Getahun, Z. *Synth. Commun*. **1985**, *15*, 907-910.
